# A Functional Promoter Polymorphism of *IFITM3* Is Associated with Susceptibility to Pediatric Tuberculosis in Han Chinese Population

**DOI:** 10.1371/journal.pone.0067816

**Published:** 2013-07-09

**Authors:** Chen Shen, Xi-rong Wu, Wei-wei Jiao, Lin Sun, Wei-xing Feng, Jing Xiao, Qing Miao, Fang Liu, Qing-qin Yin, Chen-guang Zhang, Ya-jie Guo, A-dong Shen

**Affiliations:** 1 Key Laboratory of Major Diseases in Children and National Key Discipline of Pediatrics (Capital Medical University), Ministry of Education, Beijing Pediatric Research Institute, Beijing Children's Hospital, Capital Medical University, Beijing, China; 2 Department of Cell Biology, Capital Medical University, Beijing, China; St. Petersburg Pasteur Institute, Russian Federation

## Abstract

A susceptibility locus for tuberculosis, a re-emerging infectious disease throughout the world, was previously discovered to exist on chromosome 11p15. *IFITM3* gene encoding for interferon inducible transmembrane protein 3, is located at 11p15. It acts as an effector molecule for interferon-gamma, which is essential for anti-tuberculosis immune response. In order to investigate the association between susceptibility to TB and genetic polymorphisms of the *IFITM3* core promoter, a case-control study including 368 TB patients and 794 healthy controls was performed in Han Chinese children in northern China. The rs3888188 polymorphism showed significant association with susceptibility to TB. The rs3888188 G allele, acting recessively, was more frequent in TB patients (95% confidence interval: 1.08–1.56, Bonferroni P-value: 0.039). We further assessed the effect of rs3888188 polymorphism on IFITM3 transcription in vitro. As based on luciferase promoter assays, the promoter activity of haplotypes with rs3888188 G allele was lower than that of haplotypes with rs3888188 T allele. Moreover, peripheral-blood mononuclear cells carrying rs3888188 GG genotype showed a reduced *IFITM3* mRNA level compared to cells carrying TT or GT genotype. In conclusion, rs3888188, a functional promoter polymorphism of *IFITM3*, was identified to influence the risk for pediatric TB in Han Chinese population.

## Introduction

Tuberculosis (TB),with *Mycobacterium tuberculosis* (*M. tuberculosis*) as the major pathogen, is still a major threat to worldwide human health. *M. tuberculosis* infects approximately one-third of the world's population. However, only about 10% of infected individuals eventually develop TB disease, whilst the majority of humans are naturally resistant to TB, indicating that host genetic factors play an essential role in determining TB susceptibility [1], [2]. Genetic variations in an increasing number of genes have been associated with human susceptibility to TB disease [3]–[6].

In China, the prevalence of TB in adults is 1.04‰ by WHO report 2012 [7], while the prevalence of TB in children is 0.918‰ according to a nationwide survey [8]. Compared with adults, children present a special risk group for TB due to rapid progression of a recent infection towards disease [9]–[12]. Certain pediatric TB reflects Mendelian predispositions, while adult TB seems to be more complex for genetic predisposition [11]. Studying the effects of the candidate susceptibility genes on pediatric TB may aid in the establishment of more efficient prevention of TB spread.

Interferon inducible transmembrane protein 3 (IFITM3) is a double trans-membrane protein that can be upregulated by interferons. High expression of *IFITM3* has been detected in periphery leukocytes, HeLa cell lines, and other cells and tissues [13]–[20]. IFITM3 participates in interferon-triggered processes, such as homotypic cell adhesion, anti-proliferative activities in tumor pathogenesis, and the innate immune response to virus infections [13]–[19]. Interferon gamma (IFN-γ) is produced and released in response to the presence of pathogens [21], it is secreted as a major cytokine to activate macrophages during *M. tuberculosis* infection [22]. Mutant mice lacking *Ifng* have weakened defense against *M. tuberculosis* [23], and genetic variations in *IFNG* and its receptor, *IFNGR*, have been implicated in susceptibility to human TB [24], [25]. IFITM3 has been proven to be an important component of IFN-γ signaling pathway, as down-regulation of *IFITM3* via siRNA could largely reduce the anti-virus activities conducted by IFN-γ [18]. Thus, *IFITM3* is a potential candidate gene for TB susceptibility.

The *IFITM3* gene is located on chromosome 11p15. In this regard, it is worth mentioning that Stein et al. previously found in a genome wide scan that one of the TB-linked loci was located in this chromosome region [26]. The core promoter region of human *IFITM3* is enriched with regulatory elements (such as ISRE elements, the SP1-binding motif, and other regulatory sequences), and these are thought to be critical for its transcription [27]–[30]. In this current study, we investigated the relationship between *IFITM3* and susceptibility to TB via polymorphism analysis of its core promoter region around transcription start site. To determine whether or not these *IFITM3* polymorphisms are associated with susceptibility to TB, its alleles and genotypes were analyzed in case-control study in Han Chinese pediatric population.

## Materials and Methods

### Ethics statement

Clinical investigation had been conducted according to the principles expressed in the Declaration of Helsinki. This research has been approved by the Ethics Committee of Beijing Children's Hospital. Written informed consent was obtained from the patients or the guardians of the patients that participated in this research.

### Study sample

All the participants involved in this research were Han ethnicity and originated from Northern China (Beijing city and surrounding provinces). They were vaccinated with BCG at birth, which was confirmed by the presence of a scar on the shoulder and by their vaccination records. The pediatric TB patients (n = 368) were newly diagnosed to be Pulmonary TB (PTB, pathological changes limited to in lung) or Extra-pulmonary TB (EPTB, pathological changes involving other tissues) by at least two experienced pediatricians in Beijing Children's Hospital according to the pediatric TB clinical diagnosis standard [31]–[33]. The gold standard diagnosis of pediatric TB is made when *M. tuberculosis* is isolated. There were 42 bacteriology confirmed TB patients, in addition to their clinical signs/symptoms suggestive of TB, at least 1 *M. tuberculosis* positive culture from their sputum, which could be sampled from expectorated sputum, induced sputum, nasopharyngeal aspirates, gastric aspirates, or string tests (or other relevant intrathoracic samples) were obtained. In the absence of bacteriological confirmation, the diagnosis of pediatric TB is based on a triad of (1) clinical presentation (symptoms or signs); (2) imaging (chest radiography and computed tomography scan); (3) contact history (family, close contact); (4) purified protein derivative (PPD) tuberculin skin test (using 5 TU); (5) positive clinical response to anti-TB therapy;(6)Except other lung disease, such as the various reasons pneumonia, lung tumor and so on. Clinical TB could be diagnosed if positive features of (1) plus (2) or (1) plus either two of (3)–(6) were present.

Participants of the control group (n = 794) were recruited among those admitted to Beijing Children's Hospital for surgery. All of them had negative tuberculin PPD skin-test results (<5 mm) and no history of TB or HIV infection, and were matched with TB cases for age, sex, and ethnicity.

#### DNA extraction and genotyping

Genomic DNA was extracted from peripheral leukocytes by using a Genomic DNA Extraction kit (QIAamp DNA Blood Mini Kit; Qiagen, Hilden, Germany). The core promoter of *IFITM3* was amplified by PCR using 5′-GAG CCC TGA ACC GGG ACA GTG-3′ and 5′-TGG TGT CCA GCG AAG ACC AGC-3′ primers and genotyped by sequencing using a 3730 DNA Analyzer (Applied Biosystems, Foster City, CA, USA).

#### Plasmid constructs

Forward (5′-TAT ACT GCA GCT AGC GAG CCC TGA ACC GGG ACA GTG-3′) and reverse (5′-TAT ACT GCA CTC GAG TGG TGT CCA GCG AAG ACC AGC-3′) primers were used to amplify the three most common promoter haplotypes of *IFITM3*. Promoter haplotypes were independently inserted between the NheI and XhoI restriction sites of the pGL3-based plasmid (Promega, Madison, WI, USA) to generate the pGL3-IFITM3 plasmids. Site-directed mutagenesis of the -204 G/T (rs3888188) site of the pGL3-IFITM3 plasmids was carried out using a QuickChange site-directed mutagenesis kit (Stratagene, La Jolla, CA, USA) following the manufacturer's protocol. This process generated three new constructs with the −204 G/T site changed to −204 T/G. These mutations were confirmed by DNA sequencing.

### Cell culture and luciferase assays

HeLa cells were maintained in Dulbecco's modified Eagle's medium (Gibco BRL, USA) supplemented with 10% fetal bovine serum, penicillin (100 U/ml), and streptomycin (100 U/ml). Each constructed pGL3-IFITM3 plasmid was transfected into HeLa cells with the pRL-SV40 plasmid (Promega, USA) by using Lipofectamine 2000 (Invitrogen, USA). Firefly luciferase and renilla luciferase (both via pRL-SV40 plasmid) activities were sequentially measured by a luminometer 48 h after transfection, with or without IFN-γ treatment (final concentration of 100 pg/ml and 0 h, respectively) over a serial time span (5 h and 20 h), utilizing a Dual-Luciferase reporter assay system (Promega, USA). Results were expressed as relative light units of firefly luciferase activity over relative light units of renilla luciferase activity. All experiments were performed in triplicate and repeated three times.

### Expression levels of *IFITM3* in individuals with different genotypes

A ficoll gradient density centrifugation method [14] was used for peripheral blood mononuclear cell separation. Cells were resuspended in RPMI medium with 10% fetal bovine serum. Cells (10^6^ in 0.5 ml) were seeded into each well of a 48-well plate and incubated in 5% CO2 at 37°C for 24 h with or without adding IFN-γ (final concentration of 100 pg/ml) over a serial time span (2 h, 8 h, and 24 h). Total RNA was extracted and used for cDNA synthesis. Real-time PCR was performed in an ABI7300 Sequence Detection System (Applied Biosystems, USA) using SYBRPremix Ex Taq II (TaKaRa Bio, Japan). The primers for *IFITM3* were as follows: forward: 5′-ATG AAT CAC ACT GTC CAA ACC TTC T-3′ and reverse: 5′-CTA TCC ATA GGC CTG GAA GAT CAG-3′. Primers for the control (18 S) reaction were: forward 5′-GGA AGG GCA CCA CCG GAG T-3′ and reverse 5′-TGC AGC CCC GGA CAT CAA G-3′.

### Genetic and Statistical anaylysis

Hardy-Weinberg equilibrium (HWE), Linkage disequilibrium (LD), allele frequency, genotype distribution, and haplotype analyses were performed using the SHEsis program (http://analysis.bio-x.cn/myAnalysis.php) [34]. Haplotype frequencies were inferred using the derived expectation maximization (EM) algorithm as implemented in the SHEsis software. Power and sample size calculations were performed using Quanto 1.2.4 software (http://hydra.usc.edu/gxe)[35]. Statistical analysis was carried out using the Statistical Package for Social Sciences version 13.0 (SPSS, Chicago, IL). Differences between non-contiguous variables, genotype distribution and allele frequency were tested by Fisher's exact test. Odds Ratios (OR) and 95% confidence intervals (CI) were examined by logistic regression analysis. The student's t-test was used to compare the promoter activity between haplotypes or *IFITM3* mRNA level between genotypes. All statistical hypothesis tests were two-sided with P-value <0.05 representing statistical significance. Bonferroni corrections were used for multiple tests.

## Results

### Patients and controls

The mean age was 5.8 y (SD 4.7; range, 2 months–17 y) for TB patients and 6.3 y (SD 4.0; range, 3 months–17 y) for the non-TB control subjects. The proportion of male subjects was 233/368 (63.3%) in the TB patient group, and 520/794(65.5%) in the control group. Pulmonary TB (PTB) and Extra-pulmonary TB (EPTB) were diagnosed in 151 (41.0%) cases and 217 (59.0%) cases respectively. Details are shown in Table 1.

**Table 1:** Characteristics of study population.

Characteristic	TB patients			Controls (N = 794)
	PTB(N = 151)	EPTB(N = 217)	Total TB(N = 368)	
Gender, Male/Female	93/58	140/77	233/135	520/274
Age, Mean years (SD)	6.2(4.6)	5.4(4.7)	5.8(4.7)	6.3(4.0)
Age group, years				
<4, N(%)	60(39.7)	112(51.6)	172(46.7)	302(38)
4∼6, N(%)	23(15.2)	22(10.1)	45(12.2)	133(16.7)
7∼9, N(%)	28(18.5)	27(12.4)	55(14.9)	171(21.5)
10∼12, N(%)	22(14.6)	34(15.7)	56(15.2)	135(17)
13∼17, N(%)	18(11.9)	22(10.1)	40(10.9)	53(6.7)
Positive Investigations				
BCG vaccinated	151	217	368	794
PPD skin test	151	217	368	0
Mtb Culture	16	26	42	–
Imaging	151	100	251	–
Contact history	32	82	114	–

PTB, pulmonary tuberculosis; EPTB, extra-pulmonary TB; FOB, fiberoptic bronchoscopy observations.

### Genotyping and genetic analysis

Eight single-nucleotide polymorphisms (SNPs) were genotyped in the 356 base-pair PCR products of the *IFITM3* core promoter region around transcription start site: −291 C>T (rs61876247, 291 base pairs upstream of the translation start site of *IFITM3*), −242 C>A (rs35409983), −231 C>T (rs28602580), −204 T>G (rs3888188), −188 T>C (rs6598045), −181 C>T (rs7478728), −178 C>A (rs71452596), −175 C>T (rs7479267). All these SNPs were in Hardy-Weinberg equilibrium (P>0.05) both in the TB and control groups. The genotyping results of detected SNPs are summarized in Table 2. Three SNPs had a minor allele frequency (MAF) lower than 5% in both control and TB groups: rs61876247 (3.4% and 2.6%, in TB and control groups, respectively), rs35409983 (1.4% and 0.6% respectively), and rs28602580 (1.8% and 0.6% respectively). Linkage disequilibrium coefficients (|D′|) between all SNP pairs were calculated, and the absolute LD (|D′| = 1 and *r^2^* = 1) was found among three SNPs, rs7478728, rs71452596 and rs7479267.

**Table 2:** The genotyping results of SNPs in *IFITM3* core promoter.

*Position	Rs No.	Allele/ genotype	TB (n,%)	Control (n,%)	?2	P-value	†Bonferroni P-value	OR [95% CI]
**−291 C>T**	**rs61876247**	T	25,3.4	41,2.6	1.33	0.271	2.171	1.32 [0.80–2.19]
		C	711,96.6	1547,97.4				
		TT	0,0	0,0	1.25	0.264	2.114	
		TC	25,6.8	41,5.2				
		CC	343,93.2	753,96.8				
**−242 C>A**	**rs35409983**	A	10,1.4	10,0.6	3.13	0.077	0.615	2.17 [0.90–5.25]
		C	726,98.6	1578,99.4				
		AA	0,0	0,0	3.15	0.755	6.043	
		AC	10,2.7	10,1.3				
		CC	358,97.3	784,98.7				
**−231 C>T**	**rs28602580**	C	13,1.8	10,0.6	6.63	0.010	0.080	2.84 [1.24–6.50]
		T	723,98.2	1578,99.4				
		CC	0,0	0,0	6.70	0.010	0.078	
		CT	13,3.5	10,1.3				
		TT	355,96.5	784,98.7				
**−204 T>G**	**rs3888188**	G	487,66.2	954,60.1	7.92	0.005	0.039	1.30 [1.08–1.56]
		T	249,33.8	634,39.9				
		GG	166,45.1	272,34.3	12.87	0.002	0.013	
		GT	155,42.1	410,51.6				
		TT	47,12.8	112,14.1				
**−188 T>C**	**rs6598045**	C	135,18.3	311,19.6	0.50	0.479	3.832	0.92 [0.74–1.15]
		T	601,81.7	1277,80.4				
		CC	11,0.3	24,0.3	0.69	0.709	5.672	
		CT	113,30.7	263,33.1				
		TT	244,66.3	507,63.9				
**−181 C>T**	**rs7478728**	T	607,82.5	1236,77.8	6.60	0.010	0.082	1.34 [1.07–1.68]
		C	129,17.5	352,22.2				
		TT	251,68.2	478,60.2	6.99	0.030	0.243	
		TC	105,28.5	280,35.3				
		CC	12,3.3	36,4.5				
**−178 C>A**	**rs71452596**	A	607,82.5	1236,77.8	6.60	0.010	0.082	1.34 [1.07–1.68]
		C	129,17.5	352,22.2				
		AA	251,68.2	478,60.2	6.99	0.030	0.243	
		AC	105,28.5	280,35.3				
		CC	12,3.3	36,4.5				
**−175 C>T**	**rs7479267**	T	607,82.5	1236,77.8	6.60	0.010	0.082	1.34 [1.07–1.68]
		C	129,17.5	352,22.2				
		TT	251,68.2	478,60.2	6.99	0.030	0.243	
		TC	105,28.5	280,35.3				
		CC	12,3.3	36,4.5				

Logistic regression analyses were used for calculating Odds Ratios (OR) and 95% confidence intervals (CI). χ2 and P-value was determined by Fisher's exact test. †P-value was corrected by Bonferroni's approach. *Calculated from the translation start site.

Genetic association of a SNP rs3888188 with TB disease was observed after an application of the Bonferroni correction for multiple testing. The frequency of G allele in TB group was significantly higher than that in control group (OR: 1.30, 95% CI: 1.08–1.56, Bonferroni P = 0.039). Analysis of genotypic distribution of rs3888188 using 3×2 χ2 test revealed significant difference between TB group and control group (Bonferroni P = 0.013). No statistical differences were observed between PTB and EPTB subgroups (data not shown). Further, a 2×2 χ2 test was used by combining different genotype combinations to test dominant (GG+GT versus TT) and recessive (GG versus GT+TT) models of inheritance. The OR for the G allele of rs3888188 as a possible risk factor was 1.58 (95%CI: 1.32–1.89, P = 5.30e–007) under a recessive model and 1.12 (95% CI: 0.87–1.45, P = 0.384) under a dominant model. Thus, a Mendelian recessive trait was accepted for the inheritance pattern.

As three SNPs (rs61876247, rs35409983, and rs28602580) had an MAF lower than 5% in both control and TB groups and rs7478728 alone could represent rs71452596 and rs7479267 (in absolute LD), Haplotype analysis was performed for rs3888188, rs6598045 and rs7478728. Accepting the lowest frequency threshold for this haplotype analysis to be 0.05, three haplotypes including GTT, TCT, and TTC were identified (Table 3). The frequency of GTT haplotype (carrying rs3888188 G allele) was significantly higher in TB patients than that in controls (OR: 1.31, 95%CI: 1.09–1.58, P = 0.004), while the frequency of TCT haplotype (carrying rs3888188 T allele) was significantly lower in the TB group than that in controls (OR: 0.71, 95% CI: 0.57–0.89, P = 0.003).

**Table 3:** Haplotypes carried out using rs3888188, rs6598045, and rs7478728 by SHEsis.

Haplotype	TB, N (%)	Control, N (%)	?2-value	P-value	OR [95%CI]
**G T T**	466.47(63.4%)	889.38(56%)	8.15	0.004*	1.31[1.09–1.58]
**T C T**	124.95(17%)	345.86(21.8%)	8.71	0.003*	0.71[0.57–0.89]
**T T C**	113.07(15.4%)	250.20(15.8%)	0.22	0.641	0.94[0.74–1.20]

* P-value <0.05 indicates a significant difference between TB patients and controls.

### Promoter activities

**Figure 1:**
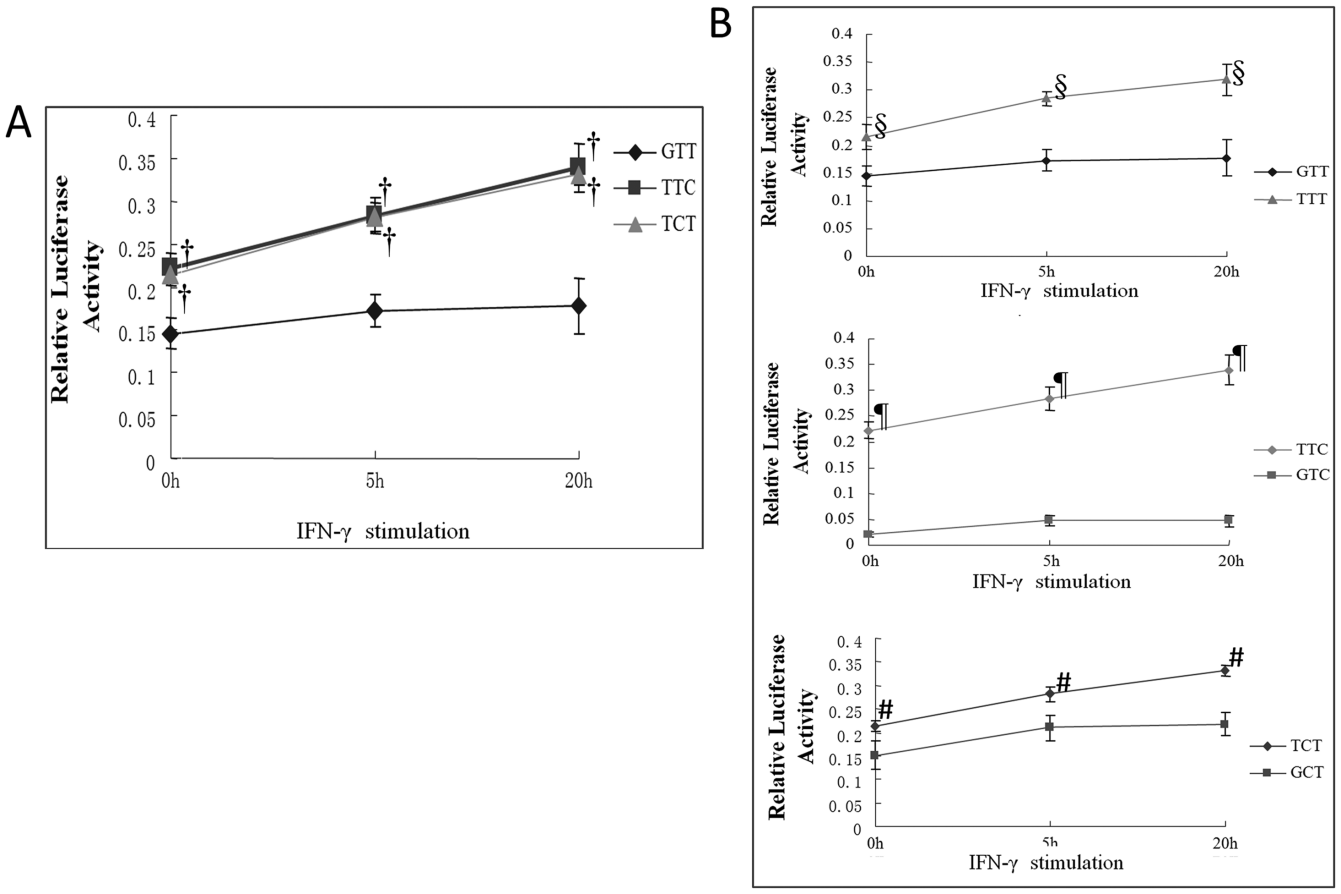
Promoter activity and gene expression analysis of *IFITM3*. Data are shown as the mean of triplicate determinations ± standard deviation (SD) and are representative of three independent experiments. Significant difference was defined as a P-value <0.05 by two-tailed unpaired t test. (A) Dual luciferase assays for haplotypes composed of rs3888188, rs6598045, and rs7478728: comparison of the relative luciferase activities of the ‘GTT,’ ‘TTC,’ and ‘TCT’ haplotypes; † indicates significant difference in comparisons of both GTT vs. TTC and CTT vs. TCT. (B) Dual luciferase assays for haplotypes before and after site-directed mutagenesis: § indicates a significant difference for GTT vs. TTT, ¶ for GTC vs. TTC, and # for GCT vs. TCT.

Three common promoter haplotypes, GTT, TTC, and TCT were amplified from genomic DNA of known homozygous individuals and subcloned into pGL3-basic plasmid construct to generate pGL3-IFITM3 plasmids. Site-directed mutagenesis was then carried out on rs3888188 SNP in each plasmid. Following transfection and subsequent dual luciferase assays, the transcriptional activity of the GTT (carrying rs3888188 G allele) haplotype promoter was significantly lower than that of TTC and TCT (both carrying rs3888188 G allele) promoter (Fig. 1A). Furthermore, the transcriptional activity of the promoter construct with the TB-susceptible rs3888188 G allele was lower than that of the rs3888188 T allele for each pair of haplotypes (before and after site-directed mutagenesis) (Fig. 1B).

### Expression levels of *IFITM3* in lymphocytes with different genotype of rs3888188

**Figure 2:**
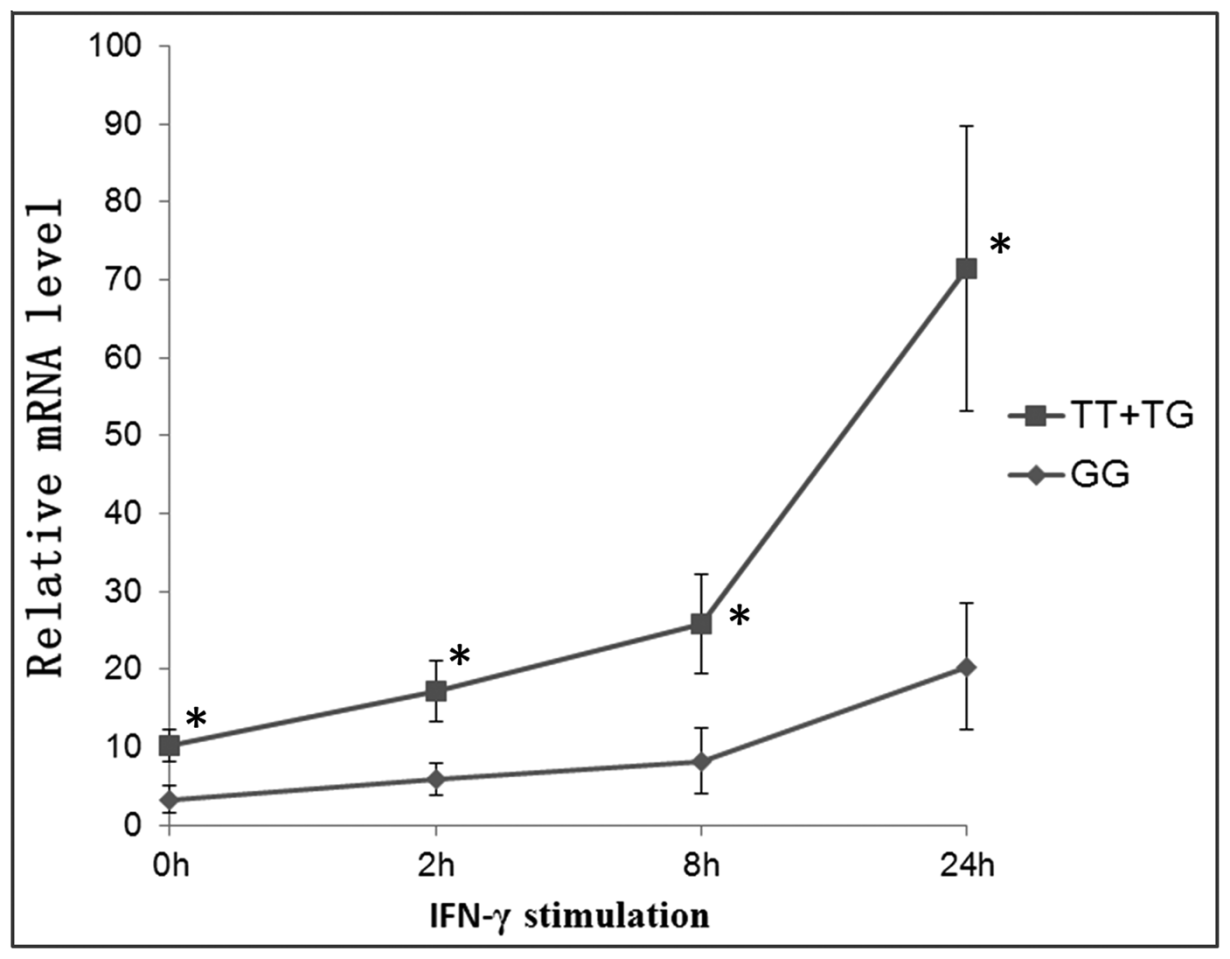
Gene expression analysis of *IFITM3*. The relative mRNA expression levels of *IFITM3* were displayed according to genotypes of rs3888188: Data are shown as the mean determinations ± standard deviation (SD) and are representative of three independent experiments. Significant difference was defined as a P-value <0.05 by two-tailed unpaired t test. * indicates a significant difference as compared to GG genotype.

As a Mendelian recessive trait was accepted for rs3888188 G allele, we further investigated whether rs3888188 GG genotype would influence *IFITM3* mRNA expression level. Fifteen healthy individuals (4 with rs3888188 TT genotype, 6 with TG genotype, and 5 with GG genotypes, age 10 ± 3 years) were recruited for this experiment. *IFITM3* mRNA analysis was performed in lymphocytes from these 15 individuals. The relative mRNA levels of *IFITM3* in all genotypes continued to rise within 2–24 hours after IFN-γ was added. Lymphocytes carrying the rs3888188 GG genotype had significantly lower *IFITM3* expression level compared to the cells carrying other genotypes (TT plus TG) (Fig. 2).

## Discussion

Children not only have an increased risk of developing progressive disease following exposure, but have a much greater risk of developing disseminated forms of the disease such as tuberculosis meningitis (TBM). Although this could be largely resulted from the immaturity of the immune response, certain pediatric TB reflects Mendelian predispositions, while adult TB seems to be more complex for genetic predisposition [11].

IFITM3 is an important component of the IFN-γ signaling pathway. This has been demonstrated through down-regulation of IFITM3 via siRNA, which reduced IFN-γ antiviral activity by 40–70% [17]. IFN-γ is thought to be essential factor acting against different infectious agents including tuberculosis. Successful immune reaction against *M. tuberculosis* requires strong IFN-γ signaling. As such, *IFITM3* is a potential candidate gene for TB susceptibility. Thus, we speculated that IFITM3 might have a clear influence on the occurrence of TB. Until now, no data on the *IFITM3* genetic polymorphisms were published for Han Chinese population. As promoter variation might influence gene expression, we initially investigated the association of polymorphisms in the core promoter region of *IFITM3* with TB in a subset of the pediatric population of Han Chinese ethnicity.

This case-control study showed the rs3888188G allele to be linked to the increased risk of TB. In genetic studies, the power of a sample set to detect an association greatly depends on the allele frequency and the size of its effect [34]. In our study, a power to detect an effect for rs3888188 was calculated to be high (99.6%) under the sample size used. The frequency of the rs3888188 G allele in TB group was significantly higher than that in the control group after Bonferroni correction and a recessive model of inheritance was found significant. Besides, the frequency of GTT haplotype (that contains rs3888188 G allele) was significantly higher in TB patients than in controls while the frequency of TTC haplotype (rs3888188 T allele) was significantly lower in TB patients than in controls. Since the *IFITM3* gene is located at 11p15.5, these findings may also support the identification of the TB-susceptibility locus at 11p15 suggested by the genome scan [26].

Because IFITM3 plays a pivotal role in IFN-γ signaling and successful immunity against *M. tuberculosis* requires strong IFN-γ signaling, we further hypothesized that the TB-susceptible rs3888188 G allele could lead to reduced *IFITM3* transcription. By dual-luciferase reporter analysis, we found the transcriptional activities of the rs3888188 G allele promoter to be lower than that of the T allele promoter. We also noticed that lymphocytes carrying the rs3888188 GG genotype had significantly lower *IFITM3* mRNA level compared with cells carrying other two genotypes (TT plus TG). These observations indicated that insufficient transcriptional activity of *IFITM3* could be a risk factor for TB susceptibility and that IFITM3 has a protective role against TB. However, further studies are needed to determine the specific transcription factors involved in the rs3888188-regulated promoter activity.

One possible mechanism by which *IFITM3* expression levels may modulate susceptibility to TB is a reduced capacity to eliminate *M. tuberculosi*s in phagolysosome. IFITM3 participates in the lysosomal pathway and was shown to limit viral replication at a late stage of endocytosis [18], as *IFITM3* overexpression lead to the expansion of enlarged acidified compartments consisting of lysosomes. Previously, lysosome related genes, such as *SLC11A1* (encoding solute carrier family 11 member 1, a lysosome membrane protein) [36] and *IRGM* (encoding immunity-related GTPase family M, mediating fusion of *M. tuberculosis*-contained endosome with lysosome) [37], were proven to be associated with TB susceptibility. Lysosomal pathway plays an important role in TB pathogenesis [36], [37], it may directly determine the successful elimination of *M. tuberculosis* by macrophages or the survival of *M. tuberculosis* within macrophages. As sufficient expression of *IFITM3* effectively restricted infection by influenza virus [16], [17], vesticular stomatitis virus [18], filoviruses, and SARS coronavirus [16], sufficient expression of *IFITM3* might also be critical in reducing survival of *M. tuberculosis*.

The present study was conducted among newly diagnosed pediatric TB patients of Han Chinese origin and healthy controls with the same ethnic background. Association linking *IFITM3* to pediatric TB is reported here for the first time and has not been previously investigated in any population.
